# Mutations in Planar Cell Polarity Gene *SCRIB* Are Associated with Spina Bifida

**DOI:** 10.1371/journal.pone.0069262

**Published:** 2013-07-26

**Authors:** Yunping Lei, Huiping Zhu, Cody Duhon, Wei Yang, M. Elizabeth Ross, Gary M. Shaw, Richard H. Finnell

**Affiliations:** 1 Dell Pediatric Research Institute, Department of Nutritional Sciences, the University of Texas at Austin, Austin, Texas, United States of America; 2 Department of Chemistry and Biochemistry, College of Natural Sciences, the University of Texas at Austin, Austin, Texas, United States of America; 3 Department of Pediatrics, Division of Neonatology, Stanford University School of Medicine, Stanford, California, United States of America; 4 Center for Neurogenetics, Brain and Mind Research Institute, Weill Cornell Medical College, New York, New York, United States of America; Pisces Therapeutics, United States of America

## Abstract

Neural tube defects (NTDs) (OMIM #182940) including anencephaly, spina bifida and craniorachischisis, are severe congenital malformations that affect 0.5–1 in 1,000 live births in the United States, with varying prevalence around the world. Mutations in planar cell polarity (PCP) genes are believed to cause a variety of NTDs in both mice and humans. *SCRIB* is a PCP-associated gene. Mice that are homozygous for the *Scrib* p.I285K and *circletail* (*Crc*) mutations, present with the most severe form of NTDs, namely craniorachischisis. A recent study reported that mutations in *SCRIB* were associated with craniorachischisis in humans, but whether *SCRIB* mutations contribute to increased spina bifida risk is still unknown. We sequenced the *SCRIB* gene in 192 infants with spina bifida and 190 healthy controls. Among the spina bifida patients, we identified five novel missense mutations that were predicted-to-be-deleterious by the PolyPhen software. Of these five mutations, three of them (p.P1043L, p.P1332L, p.L1520R) significantly affected the subcellular localization of SCRIB. In addition, we demonstrated that the craniorachischisis mouse line-*90* mutation I285K, also affected SCRIB subcellular localization. In contrast, only one novel missense mutation (p.A1257T) was detected in control samples, and it was predicted to be benign. This study demonstrated that rare deleterious mutations of *SCRIB* may contribute to the multifactorial risk for human spina bifida.

## Introduction

Neural tube defects (NTDs) are a class of human birth defects that result from a failure of embryonic neural tube closure. Failure to complete low spinal closure causes spina bifida, incomplete cranial closure results in anencephaly, while the failure of closure of the entire neural tube is a defect referred to as craniorachischisis. Worldwide, NTDs affect 0.5-2 per 1,000 live born infants, with varying prevalence across populations. Spina bifida and anencephaly are the two most common forms of NTDs, occurring in 0.5-1 per 1,000 pregnancies in the United States [1]. Many infants with spina bifida can survive, but may endure a greatly diminished quality of life.

Although genetic factors are believed to contribute in part, to the etiology of spina bifida, the elucidation of such factors has remained elusive. This is likely due to the complex inheritance pattern and the contribution of a range of environmental factors including folic acid [2]. Indeed, more than 250 genes were causally linked to NTDs in mice [3]. Interestingly, all known planar cell polarity (PCP) genes are involved in the process of neural tube closure [4]. Homozygous PCP mutations, such as *Vangl2* D255E and S464N [5,6], *Celsr1* D1040G and N1110K [7], produced a craniorachischisis phenotype in mice. When heterozygous PCP gene mutations such as *Vangl2* D255E are combined with non-PCP mutations in mice, they produce embryos with spina bifida or exencephaly [4]. In humans, mutations in PCP core genes including *VANGL2*, *FZD6*, *CELSR1*, *PRICKLE* and *DISHEVELLED*, are associated with several kind of NTDs. including spina bifida, anencephaly and craniorachischisis [4].

*SCRIB* is a PCP-associated gene in mammals [8]. It is a member of the LAP (leucine rich repeats and PSD-95/Discs Large/ ZO-1) protein family [9,10]. The LRR region and PDZ regions are important for *SCRIB* localization and stabilization at the plasma membrane [11,12]. The *SCRIB* PDZ domain also plays an important role in physical interaction with other proteins, including the core PCP gene Vangl2, which has a PDZ binding domain [13]. In *Drosophila*, homozygous *Scrib* mutations result in loss of apicobasal cell polarity and neoplastic tissue overgrowth [14]. In mice, homozygous *Scrib* mutations, such as *circletail* (3182-3183insC) [15] and line-*90* (p.I285K) [16], cause the most severe type of NTD, craniorachischisis. In humans, *SCRIB* mutations are associated with craniorachischisis [17] and several kinds of cancer [18]. It remains uncertain whether it is associated with non-craniorachischisis NTDs in human, such as spina bifida. We hypothesized that *SCRIB* mutations were associated with non-craniorachischisis NTDs, and investigated this hypothesis among infants born in California with spina bifida.

**Table 1:** Novel Rare Variants detected in NTDs in the CDS of *SCRIB* Gene in Spina Bifida.

Nucleotide change^a^	Amino Acid change	Amino Acid locate domain	Amino Acid conservation	PolyPhen prediction	SIFT prediction
c.1096 G>A	p.A366T	LRR 14	No	probably damaging	DAMAGING
c.1655 C>T	p.T552M	N/A	No	possibly damaging	Tolerant
c.3128 C>T	p.P1043L	3rd PDZ	Yes	probably damaging	DAMAGING
c.3943 G>A	p.A1315T	N/A	No	benign	Tolerant
c.3995 C>T	p.P1332L	N/A	Yes	possibly damaging	Tolerant
c.4559 T>G	P.L1520R	N/A	Yes	possibly damaging	DAMAGING

^a^ Nucleotide numbering reflects cDNA numbering with + 1 corresponding to the A of the ATG translation initiation codon 1 in the reference sequence. NCBI Reference sequence number for *SCRIB*: NM_015356.

N/A: Not Available

## Materials and Methods

### Human subjects

Data were obtained from a population-based case–control study conducted by the California Birth Defects Monitoring Program (CBDMP). The CBDMP is an active, population-based surveillance system for collecting information on infants and fetuses with congenital malformations, which has been described elsewhere [19]. Included for study were 192 singleton infants with spina bifida (cases) and 190 non-malformed infants (controls). Cases were randomly selected from all live born cases and a random sample of non-malformed control infants ascertained by the CBDMP corresponding to birth years 1994–1998. The case and control infants were linked to their newborn bloodspot. All samples were obtained with approval from the State of California Health and Welfare Agency Committee for the Protection of Human Subjects.

DNA for genotyping for this study derived from anonymous newborn bloodspots. Bloodspots are collected on all newborns in California for genetic testing purposes by the State of California. The State retains the residual, unused, portion of the bloodspot and makes these bloodspots available to approved researchers. The approval process includes detailed review by the State of California Committee for the Protection of Human Subjects (the primary IRB).

**Figure 1:**
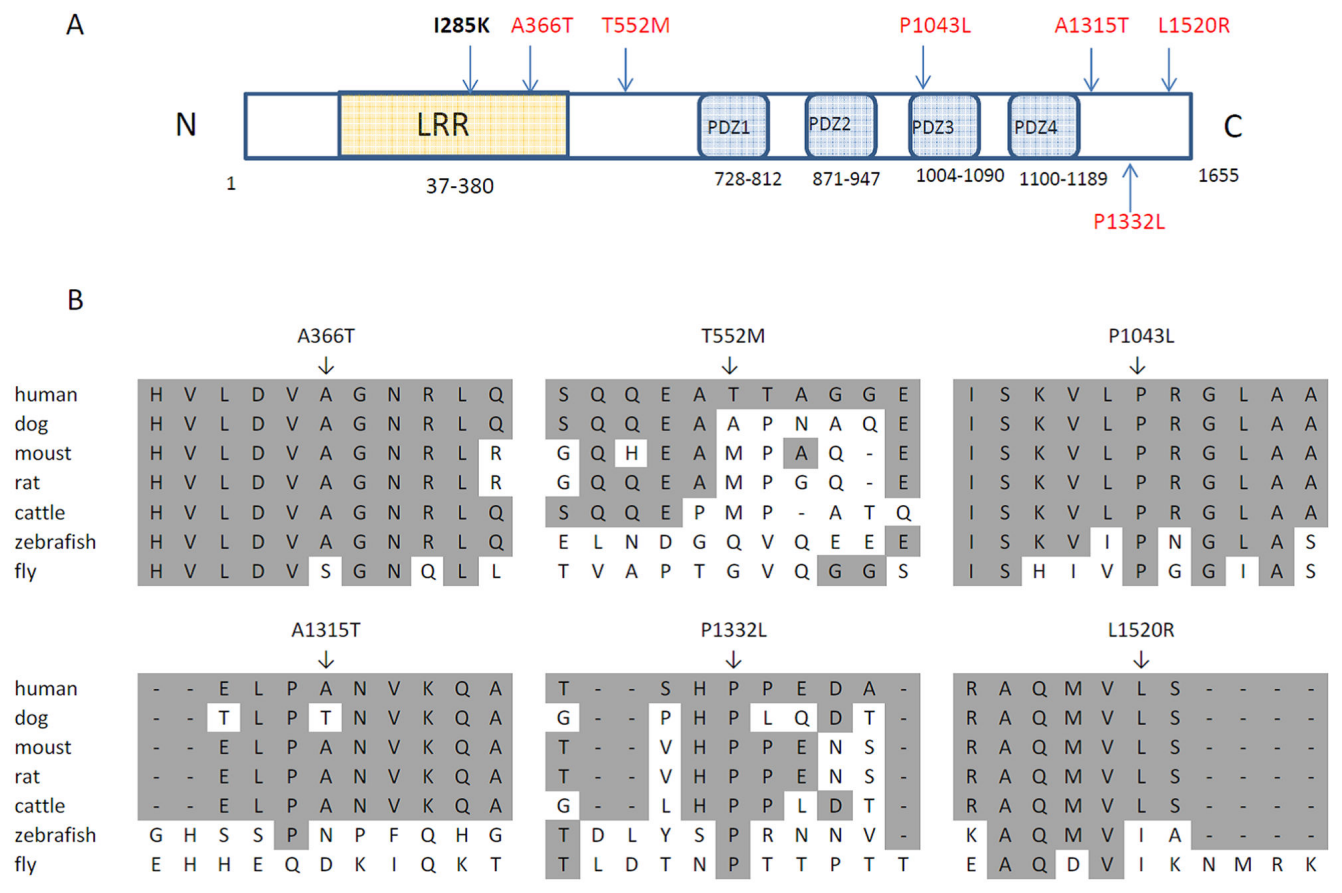
NTD specific SCRIB substitutions location and conservation analysis. (A) Location of missense changes and mouse mutations. (B) A partial alignment of SCRIB amino acid sequence between human and other vertebrates. The SCRIB variants found in NTDs affect conserved and less conserved residues. National Center for Biotechnology Information accession numbers are NP_874365.2 for human SCRIB, XP_003431867.1 for dog SCRIB, XP_003586927.1 for cattle SCRIB, NP_598850.1 for mouse Scrib, NP_001178808.1 for rat Scrib, NP_001007176.1 for zebrafish scrib and NP_001163747.1 for *Drosophila* .

The original collection of bloodspots for newborn testing includes an information form but it is not an official informed consent form. The purpose of the form is to disseminate information to the parents as to what occurs with their babies’ bloodspots and provides them with the instructions so that they can opt out and request destruction by writing to the State of California. Therefore this process is similar to the newborn genetic screening tests - "informed dissent". For the use of anonymous bloodspots for research, an "opt out" policy is applied. In other words, parents are given written materials which explain that if they do not want their child’s specimen used in research studies, they can write to the State and the State will destroy the sample. Thus, no bloodspots were used in this research project for anyone whose parents had "opted out".

### DNA sequencing

Genomic DNA was extracted from newborn screening bloodspots using the Puregene DNA Extraction Kit (Qiagen, Valencia, CA) and amplified using the GenomiPhi Kit (GE Healthcare). Coding exons and flanking exon-intron regions of the human *SCRIB* gene (NM_015356) were amplified by polymerase chain reactions (PCR) from the whole genome amplification (WGA) product. Primer sequences are available upon request. The PCR products were sequenced using the Prism Bigdye Terminator Kit (v3) on an ABI 3730XL DNA analyzer (Life Technologies, Carlsbad, CA). Both case and control samples were sequenced with either a specific forward or reverse primer. Sequencing results were analyzed using the Mutation Surveyor software V 4.0.7 (Softgenetics, Stage College, PA). The detected mutations were subsequently confirmed by a second round of whole genome amplification, PCR and sequencing analysis.

### Bioinformatics

Mutations were annotated according to the HGVS nomenclature (http://www.hgvs.org/mutnomen/). Nucleotide numbering reflects cDNA numbering with +1 corresponding to the A of the ATG translation initiation codon 1 in the reference sequence. A variant was designated as novel if it was not found in dbSNP Build 136 or in 1000 genome data (www.1000genome.org) or the Exome Variant Server (NHLBI GO Exome Sequencing Project (ESP), Seattle, WA (URL: http://evs.gs.washington.edu/EVS/) [November 2012 accessed]). Rare mutations were defined as having an allele frequency of less than 1%. The potential pathogenic effect of the missense mutations on protein function was predicted using two online programs: PolyPhen (Polymorphism Phenotyping) (http://genetics.bwh.harvard.edu/pph/) and SIFT (http://sift.jcvi.org/). CLUSTAL W program with built in Mega software (V 5.1) (http://www.megasoftware.net/) was used to analyze the conservation of *SCRIB* amino acids that were changed by the mutations. Residues were considered to be highly conserved if there was no variation in amino acid properties observed across the compared seven orthologous proteins, included five mammalian orthologus plus zebrafish and *Drosophila* .

**Table 2:** Rare variants (<1%) in the open reading frame of SCRIB that are detected in NTD patients and controls or silent, or recorded in NCBI database.

Nucleotide change	rs ID	Amino Acid change	Ct(190) /SB(192)	PolyPhen Prediction	SIFT Prediction
c.18 G>C	new	p. P6P	1/0	N/A	N/A
c.468 C>T	new	p.A1488A	0/1	N/A	N/A
c.693 G > A	new	p.R231R	0/1	N/A	N/A
c.732 G>A	new	p.L244L	0/1	N/A	N/A
c.756 G>A	rs75171224	p.Q252Q	3/3	N/A	N/A
c.762 G>A	rs144565607	p.L254L	1/0	N/A	N/A
c.1375 G>A	rs138716612	p.D459N	1/0	probably damaging	TOLERATED
c.1460 G>C	rs143419869	p. S487T	1/0	benign	TOLERATED
c.3444 C>T	rs150206819	p.D1148D	0/1	N/A	N/A
c.3555 C>T	rs142247868	p.T1185T	0/1	N/A	N/A
c.3769 G>A	new	p.A1257T	1/0	benign	Tolerant
c.3816 C>T	new	p.A1272A	0/1	N/A	N/A
c.3942 C>T	new	p.P1314P	1/0	N/A	N/A
c.3943 G>A	new	p.A1315T	1/1	benign	Tolerant
c.4437 G > A	new	p.P1479P	0/1	N/A	N/A
c.4536 C>T	new	p.D1512D	0/1	N/A	N/A
c.4662 C>T	rs111739279	p.L1554L	3/2	N/A	N/A
c.4803 C>T	rs150660931	p.P1601P	0/1	N/A	N/A

MAF: Mino Allele Frequency; Ct: Control; SB: Spina Bifida. N/A: Not done

NCBI Reference sequence number for *SCRIB*: NM_015356

### Plasmids

Human *SCRIB* full-length cDNA was cloned into pEGFP-C1 plasmid (GFP-SCRIB) and was graciously provided for our use by Dr. Patrick Humbert (Peter MacCallum Cancer Centre, Melbourne, Australia). GFP-SCRIB I285K mutant construct was acquired from Dr. Philip Stanier (UCL Institute of Child Health, London, UK). Human influenza hemagglutinin (HA) tagged VANLG2 (HA-VANGL2) plasmid was obtained from Dr. Hongyan Wang (Fudan University, Shanghai, China). *SCRIB* missense changes were introduced into GFP-SCRIB by GeneArt® Site-Directed Mutagenesis System (Life Technologies, Carlsbad, CA).

### Subcellular localization

MDCK II cell line was chosen for use in SCRIB mutation subcellular localization studies due to its epithelial characteristic and being commonly used in epithelial cell surface polarity studies. MDCK II cells were purchased from Sigma-Aldrich and cultured according to the manufacturer’s protocols. One day before transfection, cells were seeded in 4 chamber 35 mm glass bottom dishes (4x10^4^/chamber) (In Vitro Scientific, Sunnyvale, CA). Transfection was performed using Lipofectamine 2000 regent (Life Technologies, Carlsbad, CA) according to the manufacturer’s manual. Forty-eight hours later, cells were washed twice with PBS and incubated 5 minutes with Hochest 3342 (1µg/ml) (Invitrogen) and CellMask™ Plasma Membrane Stains (Life Technologies, Carlsbad, CA) (2µg/ml) solution. The cells were then washed 3 times with PBS and fixed in 4% PFA (paraformaldehyde in phosphate-buffered saline) for 10 minutes at 37^°^C, followed by 3 more PBS washes. Cells were examined and photographed by a laser scanning confocal microscope (LSM710, Leica). The transfection efficiency of GFP-SCRIB wild type and its mutant constructs was measured and calculated on the Operetta High Content Screening System (PerkinElmer).

**Table 3:** Common Variants (MAF>=0.01) in the Coding Sequence of SCRIB Gene detected in this study.

Nucleotide change	rs ID	Amino Acid change	PolyPhen Prediction	SIFT Prediction	MAF Ct/NTD	p Value
c.474 C>T	rs56748182	p.L158L	NA	NA	0.0421/0.052	0.504
c.1276 G > A	rs148555909	p.D426N	possibly damaging	TOLERATED	0.0211/0.01	0.233
c.1512 G > A	rs144626855	p. S504S	NA	NA	0.0316/0.034	0.857
c.1651 G > A	rs118022661	p.A551T	benign	TOLERATED	0.0421/0.049	0.617
c.3921 G > A	rs11784217	p.P1307P	NA	NA	0.0211/0.013	0.387
c.4098 C>T	rs72693351	p.R1366R	NA	NA	0.0263/0.042	0.291
c.4663 G > A	rs117338714	p. G1555S	benign	TOLERATED	0.05/0.047	0.836
c.4751 C>T	rs146664605	p.P1584L	benign	TOLERATED	0.0158/0.013	0.714

MAF: Mino Allele Frequency; Ct: Control; SB: Spina Bifida. NA: Not Done

NCBI Reference sequence number for *SCRIB*: NM_015356

The subcellular localization of GFP expression was classified to three different categories: (1) GFP present only at membrane; (2) GFP distributed at the membrane and in the cytoplasm; and (3) GFP exclusively in the cytoplasm. For each transfection, at least 50 cells were counted and each construct transfection was performed three times. Quantitative data between wild type and mutants were evaluated by Chi-square analysis.

**Figure 2:**
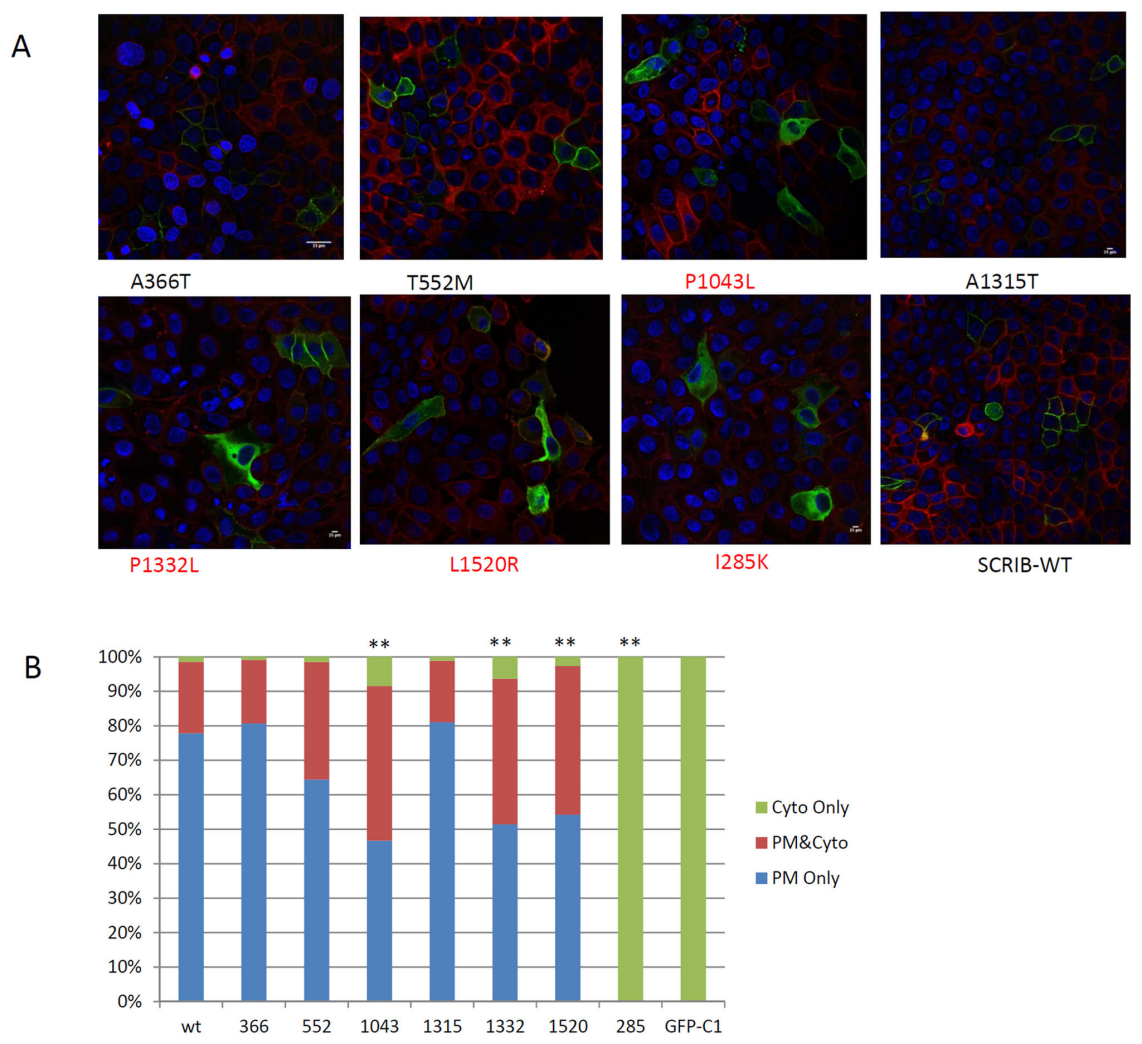
Effect of SCRIB mutations on protein subcellular localization. A: Protein localization in MDCK cells, green indicated GFP-SCRIB and its mutant, blue indicated cell nucleus. B: Quantitative analysis of protein localization for GFP-SCRIB. Cells were scored as cytoplasmic only, plasma membrane (PM) and cytoplasmic combined, or PM only. **Statistically significant differences of percentage cytoplasmic and percentage PM compared with wild-type (P < 0.001) were determined by Chi-square analysis.

### Immunoprecipitation, Immunoblotting

The HEK293T cell line was chosen in immunoprecipitation assay because of its high transfectability. In this study, the HEK293T cell line was kindly provided by Dr. Edward Marcotte (Department of Chemistry & Biochemistry, University of Texas at Austin) and was cultured in DMEM (Life Technologies, Carlsbad, CA) supplemented with 10% FBS and 2mM glutamine. One day before transfection, cells were seeded into 6-well plates (6X10^5^cells/well). GFP-SCRIB (2µg) or its relative mutant plasmids were co-transfected with HA-VANGL2 (2µg). Twenty-four hour post-transfection, cells were washed twice with ice-cold PBS and lysed with 200µl 1% NP-40 lysate buffer containing 1X cocktail protease inhibitor (Roche). We removed 30µl lysate as inputs, examined the input by anti-GFP or anti-HA immunoblotting. The remaining cell lysate was used for GFP-SCRIB and HA-VANGL2 co-immunoprecipitation. The co-immunoprecipation was performed using a Pierce HA Tag IP/Co–IP Kit (Thermo Fisher Scientific Inc.), according to the instructions. The co-precipitates were run on SDS-PAGE followed by western blot detection, immunoblotting with anti-GFP antibody for GFP-SCRIB detection and anti-HA antibody for HA-VANGL2 detection.

**Figure 3:**
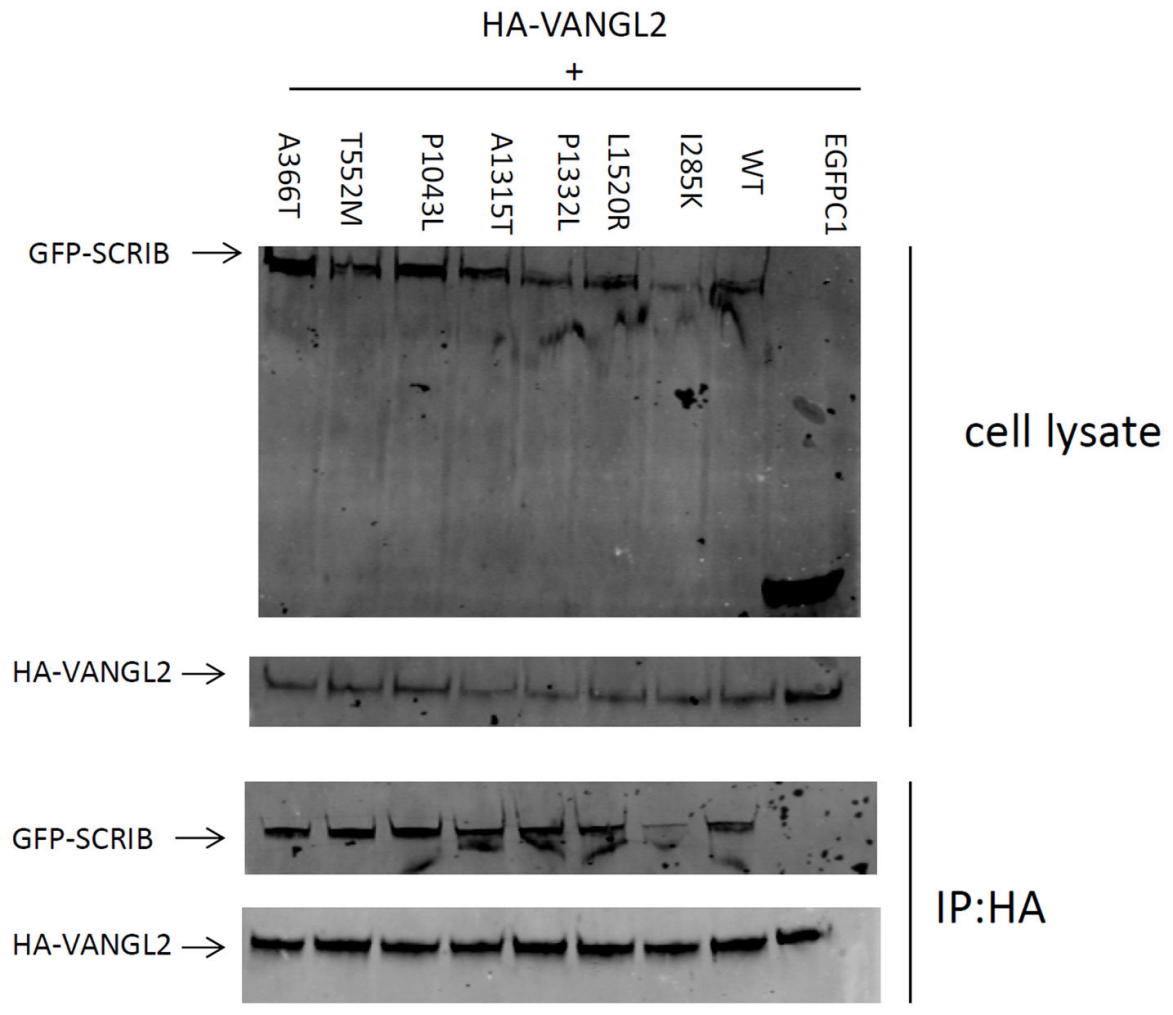
Effect of mutations on SCRIB physical interaction with VANGL2. Western blotting of the lysate with anti-GFP demonstrates the presence of wild-type GFP-SCRIB protein and mutant proteins with different missense changes. Following HA immunoprecipitation, blotting with anti-GFP confirms interaction between SCRIB and VANGL2 with all of the missense variants tested.

## Results

### Six rare missense mutations were identified in *SCRIB* in spina bifida

We detected six *SCRIB* missense mutations among 192 spina bifida infants. They were: c.1096 G > A (p.A366T); c.1655 C>T (p.T552M); c.3128 C>T (p.P1043L); c.3943G>A (p.A1315T); c.3995C>T (p.P1332L) and c.4559T>G (P.L1520R) (Table 1). None of these mutations was found in the dbSNP database or 1000 genome data, or in the 190 infant controls. p.P1332L was found in the ESP database, with a minor allele frequency of 0.007 among European American (EA) populations. Among of these six mutations, p.A366T was localized to the LRR domain, p.1043L was within the third PDZ domain, while the other four variants did not locate in these two classical domains of SCRIB (Figure 1A, Table 1). Five of the six mutations were predicted-to-be-deleterious by PolyPhen, while three of them were predicted-to-be-deleterious by SIFT. PolyPhen and SIFT each has its own advantages and disadvantages; therefore, combining the results from both programs was thought to increase the prediction sensitivity [20]. Using this strategy, five mutations were determined as “predicted-to-be-deleterious” (Table 1). During amino acid conservation analysis, three (p.P1043L, p.P1332L and p.L1520R) mutations located at highly conserved nucleotides, and the others (p.A366T, p.T552M and p.A1315T) involved less conserved nucleotides (Figure 1B). p.P1043L was identified in a case with an open defect in the thoracic area, others were found among cases with defects in the lumbosacral area. We also detected six novel synonymous mutations in case infants, but not control infants (Table 2). Eight common SNPs were identified in both cases and controls, and there were no significant differences between the groups (Table 3).

### Subcellular localization

Previous studies demonstrated that *SCRIB* must localize to the cell membrane in order to function normally [13,17]. The *SCRIB* mutation (p.I285K) which caused craniorachischisis in mice adversely affected SCRIB membrane localization [17]. We compared the SCRIB membrane localization of the six case specific missense mutations with the wild type localization serving as a positive control, and the mouse mutation p.I285K serving as a negative control. Three of the six mutations(p.P1043L, p.P1332L and p.L1520R), all of which change a highly conservative amino acid (Figure 1B), significantly increased the proportion of cells with cytosolic localization, like the mouse pathogenic line-*90* mutant (p.I285K), while the other three mutations (p.A366T, p.T552M and p.A1315T) maintained the same subcellular localization pattern as wild type (Figure 2). No significant difference in transfect efficiency was detected between GFP-SCRIB wild type and its mutant constructs (Supplementary Figures S1, S2, and S3 in File S1).

### Effect of mutant on physical interaction with VANGL2

VANGL2 is a core PCP gene that when mutated, causes craniorachischis in mice [5,6] as well as different kinds of NTDs in humans [21,22]. Of the six mutations identified, one of them (p.P1043L) was located in the third PDZ domain (PDZ3) of SCRIB, which could affect the physical interaction with PZD binding motif in VANGL2. We also performed co-immunoprecipitation of SCRIB and its mutants with VANGL2. We found that none of them lost the physical interaction between SCRIB and VANGL2 (Figure 3).

## Discussion

Homozygotes of PCP mutations and compound heterozygous mutations of two or more PCP genes are known to cause spina bifida, exencephaly and craniorachischisis in mice [23]. *SCRIB* mutations have previously been identified in craniorachischisis patients; however, it is not clear whether *SCRIB* mutations are associated with non-craniorachischisis types of NTDs in humans. We identified for the first time five predicted-to-be-deleterious mutations of which three were confirmed in functional analysis, in 192 spina bifida case infants. All of these mutations save one (p.A1315T), was found among infants born before 1998, the year when mandatory folic acid fortification started in the US. No novel predicted-to-be-deleterious mutations were found in control infants. Our data indicate that *SCRIB* mutations may underlie the pathogenesis of human spina bifida. The number of patients with spina bifida carrying novel SCRIB mutations predict to be pathogenic in this study (5 of 192; 2.6%) is comparable to the previous study (1 out of 36; 2.8%). The number of confirmed functional SCRIB mutations identified in spina bifida in this study (3 of 192; 1.6%) is less than that identified in a previous craniorachischisis study (1 out of 36; 2.8%) [17].

In the subcellular localization assay, mutations p.P1043L, p.P1332L and p.L1520R significantly altered the distribution of SCRIB subcellular localization, as more GFP-SCRIB localized to cytoplasmic instead of membrane domains. Previous structure–function analysis of human SCRIB indicated that both LRR and PDZ domains are required for correct localization [24,25]. In our study, one of the three functional mutations (p.P1043L) was located at the third PDZ domain. Prolines were highly conserved in PDZ1 domain, and were less conserved in PDZ3 and PDZ4 domain. They were not conserved in PDZ2 domain (Supplementary Figure S3 in File S1). Two of the three functional mutations (p.P1332L and p.P1520R) were not located in either the LRR or the PDZ domain. They were located close to the C terminal of SCRIB, after the fourth PDZ domain. This is similar to the previously published SCRIB mutation p.R1535Q [17]. Our data, when combined with previously published results, suggested that in addition to the LRR and PDZ domains, other regions of the SCRIB protein, especially the C terminus of SCRIB, may play an important role in human SCRIB subcellular localization.

We did not detect any obvious adverse effects of the mutations on the physical interaction of SCRIB with VANGL2. Although one of the conservative mutations (p.P1043L) was located in the third PDZ domain, which is the direct interaction partner with VANGL2 PDZ binding domain, it did not abolish the interaction between SCRIB and VANGL2. SCRIB has four PDZ domains, and both the second and third PDZ domain strongly interact with VANGL2 [13]. Although the p.P1043L mutation may affect the third PZD domain, the second PZD domain still could interact with VANGL2, hence compensating for some third PZD domain variations. Due to the quantitative limitation of western blot assay, there is the possibility that these mutations may partly affect the interaction between SCRIB and VANGL2 which could not be detected by Western blotting.

Spina bifida is a birth defect with a multi-factorial etiology. *SCRIB* mutations may interact with mutations among other non-PCP genes, or other genetic and environmental factors, and contribute to the spina bifida phenotype observed here. In the future, high-throughput next-generation sequencing technology will allow us to perform a thorough search among the whole exome or even the whole genome, to identify the additional mutations that may interact with PCP mutations.

## Supporting Information

File S1 Supporting figures. Figure S1. Transfection efficiency calculation: a. count total cells number (n) by Operetta; b. selected and counted GFP positive cells number (a). Transfection efficiency = (a/n) *100%. Figure S2. GFP-SCRIB constructs transfection efficiency. No significant difference was detected between SCRIB wild type and the mutant. Figure S3. GFP-SCRIB constructs transfected GFP-positive cells fluorescence intensity. a. individual GFP-positive cell (green dot) green fluorescence intensity, red dot indicate GFP-negative cells. b. Different GFP-SCRIB constructs GFP-positive cells average fluorescence intensity.
(DOCX)
