# Supplementary material for: Mutations in Planar Cell Polarity Gene SCRIB Are Associated with Spina Bifida
Source: PLoS One. 2013 Jul 26;8(7):e69262. doi: 10.1371/journal.pone.0069262 (PMC3724847; doi:10.1371/journal.pone.0069262)
Supplement: File S1 — Figure S1. Transfection efficiency calculation: a. count total cells number (n) by Operetta; b. selected and counted GFP positive cells number (a). Transfection efficiency = (a/n) *100%. Figure S2. GFP-SCRIB constructs transfection efficiency. No significant difference was detected between SCRIB wild type and the mutant. Figure S3. GFP-SCRIB constructs transfected GFP-positive cells fluorescence intensity. a. individual GFP-positive cell (green dot) green fluorescence intensity, red dot indicate GFP-negative cells. b. Different GFP-SCRIB constructs GFP-positive cells average fluorescence intensity. (DOCX) [file pone.0069262.s001.docx]

Supplementary Figures:

Supplementary Figure 1. Transfection efficiency calculation: a. count total cells number (n) by Operetta; b. selected and counted GFP positive cells number (a). Transfection efficiency=(a/n)*100%

Supplementary Figure 2. GFP-SCRIB constructs transfection efficiency. No significant difference was detected between SCRIB wild type and its mutant.

Supplementary Figure 3. GFP-SCRIB constructs transfected GFP-positive cells fluorescence intensity. a. individual GFP-positive cell (green dot) green fluorescence intensity, red dot indicate GFP-negative cells. b. Different GFP-SCRIB constructs GFP-positive cells average fluorescence intensity.
